# Microglia Reprogramming in Glioblastoma: Stem Cell-Derived Factors as Emerging Immunomodulators

**DOI:** 10.3390/cells15090840

**Published:** 2026-05-04

**Authors:** Zahra Amiri, Beatrice Federica Tremonti, Alessandro Corsaro, Alessandra Pattarozzi, Adriana Bajetto, Federica Barbieri, Stefano Thellung, Tullio Florio

**Affiliations:** 1 Section of Pharmacology, Department of Internal Medicine, University of Genova, Viale Benedetto XV, 2, 16132 Genova, Italy; amiri.z.uni@gmail.com (Z.A.); beatricefederica.tremonti@edu.unige.it (B.F.T.); ale.corsaro@unige.it (A.C.); alessandra.pattarozzi@unige.it (A.P.); adriana.bajetto@unige.it (A.B.); federica.barbieri@unige.it (F.B.); stefano.thellung@unige.it (S.T.); 2 IRCCS Azienda Ospedaliera Metropolitana, Largo Rosanna Benzi 10, 16132 Genova, Italy

**Keywords:** glioblastoma, tumor microenvironment, tumor-associated macrophages, microglia reprogramming, extracellular vesicles, stem cell secretome, immunosuppression, metabolic reprogramming, epigenetic reprogramming, potency assays

## Abstract

Glioblastoma (GBM) remains one of the most challenging forms of cancer to treat, despite that extensive molecular profiling is now available. Indeed, intratumoral cellular heterogeneity, receptor redundancy, and adaptive resistance through compensatory signaling limit the impact of targeted therapies. Moreover, immunotherapies also underperform: checkpoint blockade and vaccine strategies did not obtain consistent benefits in a low mutational burden, poorly immunogenic tumor microenvironment (TME) dominated by immunosuppressive myeloid cells. In this article, we provide evidence that tumor-associated macrophages (TAMs), a form of CNS resident microglia and infiltrating macrophage, derived from bone marrow, adopt a spatially and transcriptionally distinct, non-binary continuum, shaped by tumor-derived signals and niche constraints, allowing glioma cells to resist to immune and pharmaceutical therapeutics. Metabolic rewiring, including hypoxia-linked glycolytic pressure, lactate signaling, and lipid-associated programs, determine immunosuppressive outputs and restrict plasticity, while epigenetic imprinting (DNA methylation, histone modifications, and chromatin regulators) stabilizes these programs and limits access to inflammatory loci. We discuss how stem cell secretome, and extracellular vesicles (EVs) and their cargo may act as tunable autocrine/paracrine inputs that may bias microglial regulatory control. Finally, we highlight major translational confounders, including EV operational definitions, blood–brain barrier (BBB) permeability and regional exposure, inconsistent dosing units, mixed myeloid compartments, and manufacturing dependent variability. Therefore, an exposure-aware framework that integrates product identity, delivery evidence, state-sensitive potency assays, and functional endpoints would be highly desirable.

## 1. Introduction

Glioblastoma (GBM) is an aggressive primary tumor that may develop in all CNS areas with high incidence in the frontal and temporal lobes [1]. It is classified as grade 4 isocitrate dehydrogenase (IDH) wildtype diffuse astrocytoma, characterized by rapid cell proliferation and a high grade of vascularization [2]. Even though aggressive treatment has been developed, including maximal extension surgery followed by the administration of temozolomide (TMZ) and radiotherapy, overall survival (OS) is still lower than 1 year, with less than 10% of patients surviving for 5 years [3].

Moreover, although several studies based on genomic profiling and biochemical characterization have highlighted multiple possible approaches for targeted therapy, they have not yet evolved into leading therapeutic approaches for GBM. In detail, a tumor’s molecular landscape was exhaustively mapped, identifying genetic and functional alterations in the receptor tyrosine kinase (RTK) and their intracellular effectors (mainly the ERK1/2 and PI3K/Akt/mTOR axes) but this profound biological knowledge failed to translate into meaningful survival benefits.

The failure of many targeted therapies highlights that static bulk molecular profiling is fundamentally insufficient. The mere presence of a mutation does not guarantee therapeutic sensitivity, as extensive intratumoral heterogeneity and compensatory signaling networks can effectively override drug responses [4]. In fact, GBM cells show functional receptor redundancy by preserving downstream signaling through multiple co-activated RTKs, including MET and PDGFR. This co-activation enables the tumor to evade single-agent inhibition, as the blockade of a primary target like EGFR is efficiently bypassed by these alternative receptors able to activate PI3K/Akt signaling [5]. Therefore, the old “one size fits all” strategy has hit a therapeutic ceiling, failing to overcome the complex, non-genetic resistance mechanisms that characterize this disease [6].

Immune checkpoint blockers (ICBs) revolutionized the pharmacological approach to several solid tumors, such as melanoma, prompting their investigation in GBM. Nevertheless, targeting PD-1, PD-L1, or CTLA-4 in GBM has uniformly failed to demonstrate therapeutic benefit [7]. The Check Mate 498 and 548 trials confirmed the failure of anti-PD-1 monotherapy independently from the MGMT promoter’s methylation status, underscoring a fundamental biological mismatch. To elucidate the basis of these negative outcomes, increasing attention has been directed toward the unique features of the GBM TME. GBM is characterized by low tumor mutational burden, resulting in limited neo-antigen availability and, consequently, poor immunogenicity. The tumor is infiltrated by only sparse T-cell populations, whereas immunosuppressive myeloid cells predominate, defining GBM as an immunologically “cold” tumor. Given that ICB efficacy depends on reinvigorating pre-existing anti-tumor T-cell responses, such an environment is intrinsically refractory to checkpoint blockade. Moreover, the few T-cells that do infiltrate GBM frequently exhibit profound functional exhaustion. This condition is, at least in part, metabolically driven by metabolic constraints within the CNS microenvironment [8], where tumor cells and neurons compete from nutrients. This condition deprives T-cells of essential metabolic substrates, leading to bioenergetic failure and impaired effector function [9].

In search of novel therapeutic approaches, research shifted toward active immunization, hoping to bypass the cold microenvironment by making de novo T-cell responses. In particular, different anti-tumor vaccines directed against GBM cell-specific targets were developed. This strategy, however, encountered another inherent biological constraint: the redundance of cellular signaling pathways. The failure of the Phase III ACT IV trial for rindopepimut, which targeted EGFRvIII, recapitulates the risk of using single-target approaches: tumor cells adapted to the treatment can develop a mechanism of antigen escape through the downregulation of the target protein to survive [10].

When using personalized dendritic cell vaccines like DCVax-L, partial effectiveness was reported, especially in the case of patients with tumor relapse; however, the reliability of these results is limited by trial designs that depend on external controls instead of direct randomization. Basically, the efficacy of vaccination is limited by the tumor’s low mutational burden, which restricts the repertoire of high-quality neo-antigens, and profound systemic immunosuppression that is driven by the S1P1 receptor-mediated sequestration of T-cells in the bone marrow, stopping the adaptive response at its source [11]. Beyond antigen escape, GBM also employs a more fundamental mechanism: the active downregulation of MHC class I molecules, which efficiently makes GBM cells invisible to cytotoxic CD8^+^ T-cells [12]. This loss of antigen presentation, in addition to the brain’s naturally low MHC expression, creates an immunologically silent TME that even the most potent vaccine-induced T-cells cannot infiltrate [13].

## 2. Glioblastoma Microenvironment

### 2.1. The Immunosuppressive Microenvironment of GBM

The TME confers resistance to immune cell-mediated attack in GBM. It is characterized by a marked enrichment of anti-inflammatory cytokines, particularly IL-10 and TGF-β. These soluble factors, secreted by tumor-associated macrophages (TAMs) within the TME, are not just passive by-products but represent the main means used by the tumor to build up a highly immunosuppressive environment [14]. Specifically, IL-10 is a critical modulator to dampen antigen-specific immunity: while paradoxically fueling tumor proliferation and migration, it effectively converts the host’s regulatory mechanisms into tools for malignant progression [14,15,16]. Along with TGF-β, this cytokine creates a shield that neutralizes cytotoxic T-cell activity and increases the strength of the tumor’s resistance to the immune system [17].

In addition, a broad network of additional soluble mediators and extracellular matrix-associated signals that, functionally, shape the GBM microenvironment collectively sustain immune evasion, invasion, and therapeutic resistance. For example, a pro-inflammatory environment, leading to prostaglandin E2 (PGE2) production, contributes to establishing a tumor-supportive niche by suppressing effective anti-tumor immune responses and reinforcing pro-tumorigenic signaling [18]. Moreover, extracellular matrix proteins such as tenascin-C and type IV collagen can behave as active drivers of GBM progression, contributing to the development of a permissive biochemical and physical niche, which supports tumor cell migration, invasion, and cellular plasticity [19,20].

These effects are further reinforced by matrix-remodeling proteases. In particular, the destruction of extracellular matrix barriers by matrix metalloproteinases MMP-2 and MMP-9 promotes GBM cell infiltration into adjacent brain parenchyma and enhances the crosstalk between GBM cells and tumor-associated myeloid populations [21]. Together, these features indicate that the GBM microenvironment is shaped not only by immunosuppressive cytokines, but also by extracellular matrix composition and protease-dependent remodeling, which together drive malignant progression.

A defining hallmark of GBM is a myeloid-dominant TME, in which TAMs make up to 50% of the total live tumor mass, outnumbering lymphoid cells [22]. These TAMs, however, are modified by the tumor to adopt a plastic, M2-like phenotype that not only fails to restrain tumor cell growth but also actively drives malignant progression. Functional analysis indicates that once recruited, this polarized macrophage serves as the primary engine for angiogenesis, by secreting factors such as VEGF and IL-6, and brain parenchymal invasion, by the release of MMP-2 and MMP-9 [23,24,25,26]. Moreover, clinical attempts to eliminate these cells (e.g., using inhibitors of colony-stimulating factor-1 receptor (CSF-1R) expressed by TAMs) failed since their long-term inhibition triggers the release of IGF-1, which activates the IGF-1R/PI3K survival pathway in GBM cells, and causes resistance to therapy [27].

In addition to TAMs, other immunosuppressive myeloid populations, including myeloid-derived suppressor cells (MDSCs) and N2-polarized neutrophils, contribute to the glioma microenvironment by reinforcing immune evasion and supporting tumor progression [28,29].

### 2.2. Microglia Plasticity

Microglia represent the CNS-resident myeloid cells. These cells have a distinct ontogeny from bone marrow-derived macrophages (BMDMs), since they originate from erythron myeloid progenitors in the embryonic yolk sac, and colonize the brain parenchyma before the blood–brain barrier (BBB) closes [30]. Microglia is classically categorized into M1 (neurotoxic) or M2 (neuroprotective) [31]. Within the GBM microenvironment, M2 microglia are generally associated with a tumor-supportive phenotype, as they contribute to immune suppression, promote angiogenesis, facilitate tumor cell invasion, and support malignant progression [32,33]. Even though this classification does not fully capture the complexity of glioma-associated microglial states, it remains a useful framework to describe how tumor-derived signals bias resident microglia toward functions that favor GBM growth and persistence. However, recent single-cell transcriptomic analyses challenged this binary framework, uncovering that GBM-associated microglia exist along a broad highly plastic phenotypic repertoire shaped by complex environmental setting [34]. This adaptive capacity is not accidental but it is precisely coordinated by specific signaling cascades, mainly NF-κB and STAT3 [35], which, by regulating inflammatory pathways and the HIF-1α and TGF-β/SMAD axes, are responsible for GBM cell adaptation to hypoxic niches and soluble factors. GBM uses these regulatory mechanisms to enforce a persistent, tumor-supportive phenotype that leads to progression [36,37,38].

### 2.3. Stem Cells as Paracrine Immunomodulators

Cell therapy using mesenchymal (MSCs) and neural stem cells (NSCs) was initially considered for direct engraftment in regenerative medicine. However, more recent approaches redefined their utility as paracrine regulators, due to their complex secretome that actively reprograms the local immune microenvironment [39,40].

Two distinct molecular axes underpin the immunomodulatory efficacy of these stem cell populations. Immune tolerance can be induced by both the release of soluble immune effectors, like indoleamine 2,3-dioxygenase (IDO), PGE2, and nitric oxide (NO), and the secretion of extracellular vehicles (EVs) transporting bioactive microRNAs [41,42,43,44,45]. These vesicular cargos are internalized by recipient myeloid cells, where they target transcription factor such as Pknox1, effectively enforcing a phenotypic switch from a pro-inflammatory M1 to a reparative M2 anti-inflammatory profile [46], as a mechanism for wound healing. Yet, they offer a vital proof of concept for neuro-oncology with stem cells acting as dynamic biological editors. In GBM, harnessing this intrinsic plasticity could allow us to engineer a secretome that dismantles the tumor’s protective niche.

## 3. Microglia and Macrophage Plasticity in GBM

Although TAMs were historically considered a uniform population, nowadays they are classified in at least two distinct lineages with separate functional roles (Table 1). Unlike infiltrating macrophages (BMDMs) that enter from the circulation, resident microglia have a unique molecular signature defined by the expression of *P2ry12*, *Fcrls*, and *Tmem119*, which is preserved by TGF-β signaling [47,48], and primarily reflects developmental origin and lineage identity, rather than tumor-specific functional programming. In the context of malignancy, infiltrating macrophages can be immunophenotypically distinguished from this resident population by the high expression of *Itga4* (CD49d) [49]. Transcriptomic analyses show that these recruited macrophages are transcriptionally distinct and enriched for immunosuppressive and wound healing genes compared to their microglial counterparts [50].

From this mapping, it was shown that microglia are enriched in the leading edge of tumor infiltration areas, while BMDMs mainly accumulate near blood vessels and necrotic foci. Compared to microglia, blood-derived TAMs overexpress immunosuppressive cytokines, and markers of the active phagocytosis and tricarboxylic acid (TCA) cycle. Stable lineage-specific gene markers exist for macrophages, and their ontogeny is a key determinant of activation states within the glioma microenvironment [34].

Single-cell RNA sequencing studies revealed that glioma-associated microglia do not exist in a single uniform entity but rather occupy a transcriptional continuum between homeostatic (repressed) and activated (primed) functional states. In murine glioma models, Ochocka et al. identified distinct microglial clusters corresponding to homeostatic (Hom-MG) and activated (Act-MG) populations, reflecting different degrees of transcriptional engagement within the TME [51]. Furthermore, in the human brain, microglia align along a spectrum from high expression of core homeostatic genes such as *P2RY12* and *TMEM119* to reduced homeostatic identity, accompanied by increased MHC-II and lipid metabolism gene expression indicative of an antigen-presenting, metabolically active phenotype. The “repressed” versus “primed” microglia distinction reflects a tumor-driven, stepwise remodeling of resident microglia into divergent functional programs while largely preserving lineage identity [52].

### 3.1. Metabolic Reprogramming as a Driver of Myeloid Cell Plasticity

Currently, this binary classification is being challenged: single-cell marker analysis demonstrates that the classical M1/M2 dichotomy is biologically outdated in GBM. Data from human gliomas indicate that individual TAMs simultaneously co-express canonical markers of both activation states [43]. As an example, 66% of TAMs expressing the immunosuppressive M2 marker IL10 also express the pro-inflammatory M1 marker tumor necrosis factor-α (TNF-α) [34]. This transcriptional overlap was confirmed at the protein level, since flow cytometry identifies myeloid populations co-stained for both CD86 (M1) and CD206 (M2) [53]. In addition, blood-derived TAMs exhibit a distinct metabolic paradox: they upregulate M2-associated immunosuppressive cytokines yet sustain an active TCA cycle through alternative anaplerotic inputs, such as fatty acid oxidation (FAO) or metabolism-supported glutamine [54].

Although the presence of a complex continuum of the phenotypical and biological activity of TAMs (and also microglia) from inflammatory (M1) to immunosuppressive (M2) status it is now clear, as detailed above, in this review we will define M1-like conditions in which prevailing pro-inflammatory activity is detected and M2-like conditions with more evident immunosuppressive activity.

**Figure 1:**
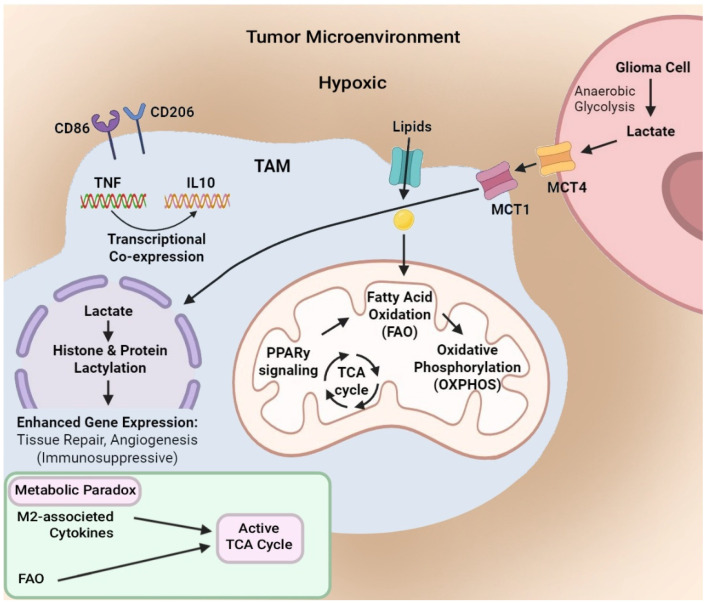
In the hypoxic GBM niche, HIF-1α preservation drives a glycolytic shift in tumor and immune cells, leading to lactate accumulation that functions as a signaling metabolite reinforcing immunosuppressive programming. Lactate is transported via monocarboxylate transporters (MCT1/4) and promotes histone and protein lactylation, enhancing expression of genes involved in tissue repair and angiogenesis, thereby biasing myeloid cells toward a tumor-supportive M2-like phenotype. In parallel, lipid availability and PPARγ-associated signaling promote FAO, sustaining OXPHOS and an active TCA cycle. Together, hypoxia-linked lactate signaling and lipid metabolism support a self-reinforcing, tumor-supportive TAM state consistent with the “metabolic paradox,” where M2-like-associated outputs coexist with maintained mitochondrial metabolism.

Metabolic reprogramming is a major mechanism by which macrophages adopt a dynamic spectrum of phenotypes, allowing them to adapt to the TME and sustain tumor progression [55] (Figure 1). Hypoxia, a major feature of the glioma niche, preserves HIF-1α expression and drives a glycolytic shift in both tumor and immune cells [56,57]. The resulting accumulation of lactate acts as a signaling metabolite that reinforces immunosuppressive programming [58,59,60]. Through histone and protein lactylation, lactate enhances the expression of genes supporting tissue repair and angiogenesis, thereby biasing myeloid cells toward a tumor-supportive M2-like phenotype [61,62].

Glioma-associated macrophages, as well as TAMs from other tumors, exhibit enhanced lipid uptake via CD36 and activation of FAO through PPARγ-associated signaling, fueling mitochondrial oxidative phosphorylation and the TCA cycle [63,64].

This lipid-driven metabolism further sustains their anti-inflammatory profile and limits plasticity, preventing repolarization to a pro-inflammatory phenotype. These metabolic alterations create a self-reinforcing circuit where hypoxia, lactate, and lipid metabolism support immunosuppressive TAMs. Preclinical studies show that inhibiting lactate transporters (MCT1/4), FAO, or PPARγ signaling can partially reverse this metabolic constraint, restoring antigen presentation and anti-tumor functions in glioma models [65,66,67,68,69,70].

### 3.2. Epigenetic Imprinting and Stabilization of Immunosuppressive Phenotypes

Epigenetic regulation plays a central role in defining macrophage polarization, linking environmental signals to stable transcriptional programs [71]. DNA methylation can contribute to the silencing of pro-inflammatory genes by preventing transcription factor binding to CpG-rich promoters such as *IL12B* and *NOS2*, thereby restraining M1-like type activation [72]. In contrast, permissive histone acetylation and methylation marks, including *H3K4me3* and *H3K9ac*, promote open chromatin and transcriptional activity, while repressive modifications such as *H3K27me3* silence inflammatory loci [73,74,75].

Enzymes such as histone deacetylases (HDACs), methyltransferases (EZH2), and demethylases (JMJD3) regulate these opposing situations, coordinating macrophage adaptation to tumor-derived stimuli [76,77]. For instance, JMJD3-mediated removal of *H3K27me3* is essential for M2-like polarization, whereas HDAC3 and EZH2 shape macrophage polarization through context-dependent regulation of promoter in chromatin, and HDAC3 is required for the induction of a substantial fraction of LPS-responsive inflammatory genes [78]. These pathways show how transient environmental signals are encoded into lasting chromatin that stabilizes either inflammatory or immunosuppressive macrophage phenotypes.

In the glioma microenvironment, tumor-derived metabolites and cytokines actively remodel the epigenetic landscape of myeloid and immune cells. Lactate functions as a potent signaling molecule that induces histone and non-histone lactylation, thereby linking metabolic shifts to transcriptional regulation [59,60,79]. Elevated lactate levels, characteristic of the Warburg effect, promote immune suppression by stabilizing HIF-1α and enhancing the expression of genes involved in angiogenesis and tissue repair [80,81]. Histone H3K18 lactylation, for instance, drives the transition of macrophages toward an M2-like phenotype, sustaining an immunosuppressive TME [62].

**Figure 2:**
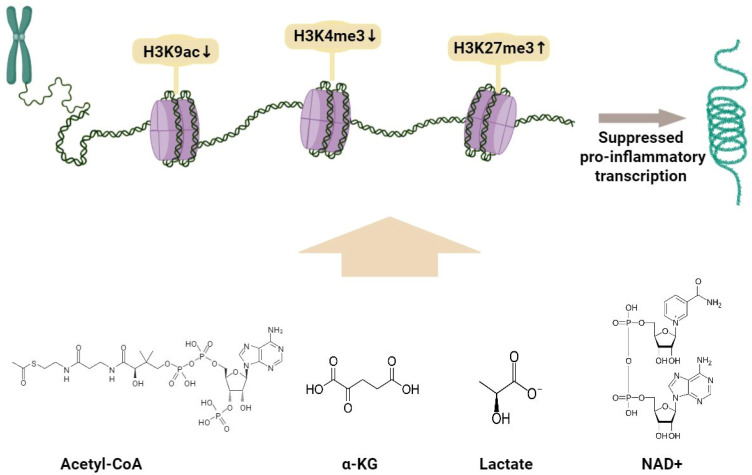
Metabolic cofactors (acetyl-CoA, α-KG, lactate, and NAD^+^) are depicted as upstream inputs that modulate chromatin regulation in TAMs. The schematic shows reduced permissive marks together with increased repressive marking (H3K27me3 ↑), consistent with chromatin compaction and diminished promoter accessibility. This epigenetic configuration is presented as a mechanism contributing to suppressed pro-inflammatory transcription in the GBM TME.

Glioma-secreted factors, such as TGF-β and IL-10, h activate or engage histone-modifying enzymes, including p300/CBP, which cooperate with lactate-driven acetyl and lactyl marks to favor chromatin accessibility at anti-inflammatory loci. In addition, metabolic intermediates such as acetyl-CoA, α-ketoglutarate (α-KG), and NAD^+^ act as essential cofactors for histone acetyltransferases, demethylases, and sirtuins, forming a biochemical bridge between metabolism and epigenetic memory [82,83,84,85]. These links indicate how metabolic rewiring in GBM translates into stable epigenetic programs that boost immune tolerance and tumor persistence (Figure 2).

Stable DNA methylation patterns and histone modifications restrict chromatin accessibility at pro-inflammatory loci while favoring permissive architecture at anti-inflammatory genes, allowing immunosuppressive transcriptional profiles to be maintained even in the absence of continuous tumor-derived signaling [86,87,88]. This epigenetic regulation provides an explanation for the limited efficacy of conventional immune-activating strategies, as inflammatory transcription factors are unable to access repressed chromatin regions despite appropriate stimulation [89]. Pharmacological targeting of chromatin regulators, including histone deacetylases, DNA methyltransferases, and bromodomain containing proteins, has been shown to activate silenced chromatin and partially restore pro-inflammatory gene expression in tumor-associated macrophages [90,91]. Therapeutic strategies aimed at active epigenetic reprogramming and supporting the exploration of RNA-based interventions, including EV-mediated miRNA delivery, as complementary approaches to overcome durable immunosuppressive programming in the glioma microenvironment are required [77,92,93,94,95].

Ontogeny sets the starting conditions, while the GBM niche determines the operative phenotype. Metabolic programming shapes transcriptional outputs, and epigenetic regulation stabilizes them, so plasticity exists but remains constrained by lineage history and local context. TAMs are not limited to a single tumor-supportive role; therefore, targeting the mechanisms that sustain this condition can help redirect activity toward anti-tumor programs. This perspective favors reprogramming-oriented strategies over simple depletion, preserving essential myeloid contributions while shifting the balance of immune function in glioma. Therefore, understanding TAM behavior requires giving attention to the paracrine signals within the tumor niche.

Microglia-specific reprogramming in GBM comes from lineage-resolved studies showing that resident microglia are not simply diluted by infiltrating macrophages but remain a distinct compartment with their own spatial distribution and transcriptional behavior inside the tumor. At the phenotypic level, GBM-educated microglia do not remain in a fixed homeostatic state, but shift toward heterogeneous activation programs, including primed, phagocytic, and dendritic cell-like states, while still retaining features of resident microglial identity [34,51]. There is evidence that specific tumor-derived stimuli are linked to these changes. GBM-derived M-CSF induces microglial release of IGFBP-1 and thereby promotes angiogenesis, while direct glioma–microglia interaction can increase microglial MMP-2 activity, enhancing extracellular matrix degradation and tumor invasion [26,96]. In parallel, glioma stem cells have been shown to drive immunosuppressive programming in macrophage/microglia populations [33], and complementary work in GBM models has linked microglial immunosuppressive remodeling to mTOR-dependent regulation of STAT3 and NF-κB signaling [97]. Tumor-derived EVs further contribute to this process by transferring regulatory cargo, including miR-21, into microglia and altering downstream gene expression to support the tumor-permissive microenvironment [98]. Together, these findings demonstrate that GBM induces a genuine reprogramming of resident microglia, characterized by defined inducing signals, measurable phenotypic remodeling, and functional consequences that contribute to angiogenesis, invasion, and immunosuppression.

## 4. Stem Cell Secretome as a Paracrine Immunomodulatory System

In the recent decades, several studies have addressed the influence of mesenchymal stem cells (MCSs) on tumor growth, with therapeutic purposes. These *s*tem cell-related therapies, developed in different pathological conditions, are often aimed at assessing the effects of the MSC secretome rather than the cells’ ability to differentiate into the tissue cell counterparts. In fact, when implanted, MSCs do not display a prolonged survival and their effects are thought to be largely mediated by paracrine humoral effects [25,99]. The MSC secretome is composed by a large set of factors, including soluble proteins, free nucleic acids, lipids, and extracellular vesicles (apoptotic bodies, microparticles, and exosomes) [100]. Recent studies have focused on the secretome, including either conditioned medium (CM) or EVs, as practical a cell-free product for translational research [101]. While CM constitutes the secretome’s entire released milieu, its soluble factors can be parted from the microvesicle fraction through separation methods like centrifugation, filtration, polymer precipitation, and size exclusion chromatography [102,103].

Within the mixture of immunoregulatory mediators, the main components include pro-inflammatory cytokines and chemokines such as TGFβ1 [104], IL-13 [105], IL1β [104], IL-6 [106,107], CXCL8 [108], and IL9 [109], along with growth and trophic factors such as VEGF and BDNF [104,110], although this is only a partial list.

The secretion of EVs provides an additional route for intercellular communication [111]. Exosomes, which are extruded when multivesicular bodies fuse with the plasma membrane, carry nucleic acids (including mRNA, miRNA, and DNA fragments), proteins, and genetic material and can contribute to cell-to-cell communication via cargo delivery to recipient cells. EVs belong to secretome-derived products, and have been linked to immunomodulatory, pro-survival, and anti-apoptotic effects, as well as angiogenesis-related readouts; however, in cancer settings, the overall outcome is highly context dependent [111,112,113].

In parallel with the immunomodulatory potential of non-tumoral stem cell secretomes, glioma stem cells (GSCs) themselves play a central role in shaping the myeloid compartment of the glioblastoma microenvironment. Through the release of soluble factors, including sCSF-1, TGF-β1, and macrophage inhibitory cytokine-1 (MIC-1), as well as EV-associated signals, GSCs recruit and reprogram microglia, stromal stem cells, and macrophages toward immunosuppressive, tumor-supportive phenotypes [110,114]. In turn, these reprogrammed myeloid cells reinforce immune evasion, support angiogenesis and invasion, and help sustain a niche that promotes GBM growth and progression [33,114,115,116]. Moreover, the expression of chemokine receptors in GBM cells provides direct support to cell proliferation and invasion since GBM cells release several chemokines (mainly CXCL12) which act as autocrine/paracrine factors on both GSCs and non-stem GBM cells [114,117].

Importantly MSC composition and functional output vary according to MSC origin and environmental context. This is one of the main reasons why different studies report divergent outcomes in GBM models [99,118], and the relative balance of these components has been proposed to influence the net biological effect of MSCs [119,120]. In the GBM context, MSCs show different effects on co-cultured glioma cells, depending on their derivation [99]. For example, proteomic profiling of the UC-MSC secretome highlighted pathway enrichment consistent with growth and angiogenesis signaling (e.g., Wnt/PDGF/VEGF), while other studies emphasize a prominent role for specific soluble factors (including TGF-β) in defined MSC GBM pairings [121,122]. MSC-derived EVs can mediate cell-to-cell communication and deliver defined miRNAs in anti-glioma strategies (e.g., miR-124/miR-124a, and miR-146b). However, since EV yield and cargo depend on pre-analytical and culture variables, signature claims should be interpreted using MISEV2018 guidelines that recommend rigorous reporting of EV isolation methods, particle and protein quantification, and the use of EV-enriched and EV-depleted markers to support claims of EV identity and cargo association [123].

The NSC secretome is enriched in neurotrophic factors such as NGF, BDNF, CNTF, and GDNF, which are commonly implicated in neural repair and homeostatic support, rather than the broad immunosuppressive cytokine repertoire more commonly highlighted for MSC secretome [124,125]. This becomes especially relevant at the EV levels in glioma models: NSC-derived exosomes have been leveraged as carriers of miR-124-3p, and transfer of this cargo has been associated with quantifiable anti-tumor readouts. This allowed the identification of miR-124 as a useful example of CNS-relevant NSC-EV cargo, alongside the neurotrophic factors (NGF, BDNF, CNTF, and GDNF) that are typically used to describe NSC secretome biology in neural repair and homeostasis [126,127].

Considering the iPSC-derived secretomes, their potential is more recognized, but their translational performance is not yet fully established. They are of therapeutic interest in their scalable production and modulation by differentiation stage and culture conditions, yet this flexibility increases heterogeneity and variability of response [128,129,130]. A proteomic-based study reported donor-dependent differences in the iPSC secretome that correlated with differences in reparative potential, which poses a barrier to clinical implementation because it challenges standardization and consistency across preparations. Focusing on EVs, undifferentiated iPSC-derived EVs have shown anti-inflammatory and pro-vascularization activity in a diabetic wound healing model, but it is still to be determined how robust these effects are across systems and what manufacturing controls are needed to make results comparable between labs [131,132,133,134]. The main issues in different stem cell secretomes are reported in Table 2.

Taking all these data together, the identification of stem cell source-dependent differences in secretome and EV activities suggests that distinct therapeutic uses may be identified for each secretome type in GBM. The MSC-derived secretome appears strongly positioned to induce broad immunomodulation, owing to its richer repertoire of soluble immunoregulatory mediators and EV-associated miRNAs. On the other hand, this complexity may also increase the unevenness in activity and context-dependent effects. By contrast, the NSC-derived secretome is mainly characterized by neurotrophic support and CNS-relevant signaling [135,136], which may offer advantages in addressing the neuroinflammatory and tissue-damaging effects of GBM, although its direct immunomodulation may be narrower than that of MSC-derived products. Finally, the iPSC-derived secretome offers the advantage of scalability and engineering flexibility, but its translational value remains limited by donor- and differentiation-dependent heterogeneity [137]. Overall, these differences indicate that secretome source selection should be not only guided by availability, but also by the dominant therapeutic goal, whether it is broad myeloid immunomodulation, neural support, or product standardization and manufacturability.

### 4.1. Mechanistic Basis and Translational Pathways

Across CNS disease models, studies on the secretome and EVs consistently converge on a common principle: microglial activation is dynamic and responsive to extracellular signals. In stroke, EV-based interventions have been shown to be related to lower inflammatory readouts and better outcomes in vivo. Song et al. showed that M2-like microglia release neuroprotective exosomes after transient ischemia, and the effect was partly mediated by miR-124 [138]. Complementary studies used stem cell EVs in cerebral ischemia–reperfusion models, while MSC-derived exosomes were reported to reduce injury and modulate microglia-related endpoints mediated by exosomal miRNA [139].

In multiple sclerosis models, the same “microglia-state” theme shows up very clearly. In an experimental autoimmune encephalomyelitis (EAE) rat model, bone marrow MSC exosomes reduced inflammatory infiltration and demyelination and were aligned with a shift toward M2-like-associated cytokines (IL-10 and TGF-β) with decreased M1-like-associated signals, consistent with microglia polarization changes as part of the therapeutic readout [140,141,142]. In traumatic CNS injury, NSC-EVs have also been shown to reduce early inflammatory cascades [143]. NSC-EV treatment after spinal cord injury reduced microglia activation and neuroinflammation, with these effects related to autophagy induction [144]. These results do not prove what will happen in GBM, but they provide a solid rationale for potential effects in tumor setting, considering that microglia can reprogram into different activation pathways in vivo, so it is reasonable to ask whether secretome or EV signals can be shaped to favor anti-tumor microglial states in the glioma microenvironment.

### 4.2. Translational Implications

Pre-analytical variables and culture conditions influence EV recovery and composition, and these variables have to be reported to allow study comparison. For EV-based therapeutics, quality control (QC) assays should follow established biologics practice, with defined specifications for identity, purity, sterility, potency, stability, and batch-to-batch consistency. Potency should be predefined as a critical quality attribute and quantified using fit-for-purpose assays that reflect the intended mechanism and biological effect [145].

A second persistent challenge is dosing. Studies variously report dose as particle number, total protein, or equivalent volume, but these units are not directly comparable, particularly once the route of administration and effective exposure are taken into account [146]. For this reason, recent translational discussions stress the need for a clear dosing rationale developed in parallel with potency readouts and manufacturing decisions. Finally, scale-up is increasingly seen as a scientific challenge, not just an engineering step, and several studies now compare EV production platforms to reach therapeutic yields while keeping product quality intact.

Another important issue in the potential pharmacological use of stem cell-derived secretomes and/or EVs, is the possibility that, in addition to possible therapeutic immunomodulatory signals, they may also deliver undesirable biological activity. Because these preparations include heterogeneous soluble factors and vesicular cargoes, they may contribute to excessive immunosuppression, vascular support, pro-survival signaling, and resistance to cell death, in a context-dependent manner [147]. In particular, the MSC secretome has been reported to contain mediators such as TGF-β1, IL-6, CXCL8, and VEGF, and some MSC secretome profiles are enriched in modulators of Wnt, PDGF, VEGF, and other related pathways, all of which may support tumor-permissive remodeling and angiogenesis [148,149]. EV preparations are associated with immunomodulatory, pro-survival, anti-apoptotic, and angiogenesis-related readouts, suggesting that their safety cannot be assumed simply from the identity of the vesicles. Therefore, translational development of both the secretome and EVs should include a dedicated safety assessment, in parallel with identity and potency testing, to exclude unintended immunosuppressive and pro-angiogenic effects, resulting in glioma-supportive activities [150,151,152].

## 5. Extracellular Vesicles as Immunomodulatory Nanocarriers in GBM

A recurring problem in the EV field is that the biophysical similarity of the particles does not equal biogenesis. Many isolates contain small vesicles with overlapping sizes, densities, and marker profiles, so the label “exosome” is rarely justified unless the biogenesis route is directly supported. Current community guidance therefore recommends using operational terms (e.g., small EVs and EV-enriched fraction) [153].

The MISEV2018 guidelines established baseline criteria for the reporting of separation methods, marker panels, and functional attribution, and MISEV2023 expands this with updated approaches and explicit discussion of limitations and advanced analytics. The relevance of these documents resides in the necessity to define which EVs are used in microglia reprogramming [154]. In a clinical setting, this precision is even more relevant and mandatory, as stated by the ISEV clinical trial position paper, which emphasizes that possible therapeutic use pushes EV studies into a regulatory mindset where identity, comparability, and risk framing become unavoidable [155].

Following systemic administration, early bioavailability of EVs is heavily influenced by innate clearance mechanisms. Accumulating evidence indicates that EVs undergo opsonization and subsequent capture by the mononuclear phagocyte system with complement activity, favoring clearance after hepatic and splenic sequestration [146,156]. An additional variable in these studies is the protein corona, which forms quickly in biological fluids and reshapes how vesicles engage with cells. Recent studies on MSC EVs show that differences in corona composition shifts preferences in cellular uptake and in vivo kinetics, and diverse EV preparations may behave differently depending on the circulating environment they encounter [157,158].

In GBM, this is particularly relevant, as microglia-derived effects cannot be meaningfully interpreted if the administered material fails to reach the CNS or is sequestered by peripheral phagocytic compartments [158]. Delivery should therefore be considered a first-order variable rather than a secondary concern.

### 5.1. Reaching the Glioma Niche: Delivery as a First-Order Variable

In GBM, BBB dysfunction often occurs but it is highly variable across brain areas, including regions showing increased permeability, and others with intact or partially intact structure and functioning, which can shelter infiltrative tumor compartments, making drug exposure spatially uneven. Patient-facing studies also highlight substantial heterogeneity within and across individuals [159,160,161].

This heterogeneity makes EV delivery a complex experimental variable. Even when EVs are detectable in the CNS after administration, their access is unlikely to be uniform across tumor regions due to local differences in permeability. As a result, any measured change in microglia-associated markers can reflect where EVs are able to reach, rather than a generalized reprogramming effect [51,162].

This becomes especially important when readouts are obtained from bulk tissue or pooled immune fractions. If both exposed and unexposed regions are analyzed together, region-specific effects may be diluted or misrepresented, and null results may simply indicate insufficient exposure in the relevant compartments. Positive changes may overestimate the breadth of microglial modulation if they arise from a subset of better-perfused areas [163,164].

Therefore, delivery approaches should be treated as a first-order confounder in EV microglia studies in GBM. Claims of microglial state change are strongest when paired with evidence of exposure in the same experimental context, ideally with region-aware assessment rather than assuming that BBB disruption implies uniform access [165].

### 5.2. Mechanism-Linked Potency Assessment

For EV-based strategies aimed at microglia modulation, biological activity cannot be assumed from exposure, uptake, or general cellular responses [166,167]. Measures such as EV internalization, changes in cell survival, or bulk cytokine shifts describe interaction, but they do not establish which microglial state has been durably altered to affect disease progression in a relevant manner [168].

Accordingly, potency assessment should prioritize readouts that reflect the persistence and directionality of microglial state changes [169]. Rather than relying on single markers or acute responses, informative assays must capture coordinated shifts in signaling outputs that align with predefined regulatory axes relevant to microglial plasticity, such as STAT-3-, NF-κB-, or HIF-1α-associated programs, without reiterating their upstream biology [170,171]. The emphasis is not on the activation pathway, but on whether these nodes converge toward a stable functional outcome.

Based on these considerations, a practical potency framework should combine lineage-aware identification of the responding myeloid populations, integrated state markers, mechanism-linked signaling readouts, and GBM-relevant functional assays [172,173]. Ideally, potency assessment should distinguish resident microglia from infiltrating macrophages, and link changes in myeloid state to the impairment of relevant GBM biological functions, including phagocytosis, cytokine secretion, antigen presenting-associated features, and effects on tumor cell proliferation and invasion [174,175].

As a practical minimum, a proposed state-sensitive potency panel could include lineage markers, distinguishing resident microglia from infiltrating macrophages, coordinated markers of inflammatory or immunosuppressive state, mechanism-linked signaling outputs, and GBM-relevant functional assays. In this context, informative assessment may include P2RY12 and TMEM119 for resident microglia, pathway-linked outputs related to STAT3, NF-κB, and HIF-1α activity, and functional readouts such as phagocytosis, cytokine secretion, antigen-presentation-associated features, and effects on tumor cell invasion [176,177,178,179].

Mechanisms linked to potency assays also provide important interpretive information. EV preparations, similar as far as physical or compositional metrics, may substantially diverge in their ability to induce sustained microglial modulation [180]. Without state-sensitive assays, negative findings can be considered to be caused by biological inefficacy, while the results may instead reflect mismatched readouts; on the contrary, positive effects may be overstated if they capture only short-term perturbations [145,181].

Embedding potency definitions, explicitly tied to durable microglial state changes, strengthen the connection between exploratory observations and translational intent. This approach limits overinterpretation, clarifies failure events, and provides a rational basis for comparing EV preparations beyond descriptive or exposure-based metrics [145,182].

### 5.3. Dose and Effective Exposure

Defining EV dose is another persistent source of inconsistency among EV-based studies. Particle number, total protein content, and equivalent volume have all been used as dosing units, yet these measures are not interchangeable and do not scale uniformly with the biological effects. Consequently, nominal dose equivalence across studies often masks substantial differences in the amount of biologically active material really delivered [183,184,185]. This ambiguity becomes particularly problematic when dose is interpreted independently of effective exposure. Identical particle counts may correspond to markedly different functional payloads depending on EV composition, cargo enrichment, and preparation method [186]. Protein-based dosing can be skewed by co-isolated non-vesicular components, obscuring the relationship between administered material and microglial engagement. Without anchoring dose to biological response, comparisons across studies remain largely descriptive [183,185,187].

The route of administration further decouples administered dose from effective CNS exposure. Systemic delivery introduces variability that is difficult to quantify, while local delivery alters exposure kinetics without guaranteeing broader distribution. Dose–response relationships derived from peripheral or in vitro systems cannot be assumed to translate directly to the glioma context. Effective exposure, rather than nominal dose, becomes the relevant variable for interpreting biological outcomes.

To improve comparability among preparations, it would be relevant to refer to EV dose not only as a particle number, protein amount, or administered volume, but also in relation to biological potency. In this context, a more informative framework may combine a physical dosing metric with a potency-linked unit under standardized assay conditions. This approach would help distinguish nominal dose from biologically effective dose and improve comparison across studies using different preparations, delivery routes, and manufacturing methods [188].

Existing reports illustrate how EV activity can be assessed using measurable functional readouts. Pachler et al. established a dual in vitro immunomodulatory potency assay in which stromal cell-derived EV activity was quantified through dose-dependent inhibition of phytohemagglutinin-induced T-cell proliferation, while also showing that the same EV preparations did not reproducibly suppress alloantigen-driven mixed leukocyte reactions. In a more translational setting [189], Labusek et al. assessed the in vitro immunomodulatory function of clonally expanded immortalized MSC (ciMSC)-EV and primary MSC-EV preparations using a multi-donor mixed lymphocyte reaction assay, in which a defined EV protein dose was evaluated for its effect on T-cell activation. Together, these studies illustrate that EV potency can be anchored to predefined functional immune readouts rather than inferred solely from particle number, protein content, or administered volume [190]. For microglia-focused applications, dose definition should therefore be aligned with potency readouts and delivery constraints. A meaningful dose is one that produces a reproducible mechanism-consistent change in the microglial state under defined exposure conditions. When dosing is disconnected from both exposure and functional outcome, negative findings may reflect underexposure rather than a lack of efficacy, while positive effects may not predictably scale.

Addressing these issues requires treating dose as an integrated parameter rather than a standalone metric. Explicit linkage between administered amount, effective exposure, and measured biological response will improve interpretability and reduce ambiguity when EV-based immunomodulatory strategies are evaluated across experimental systems and developmental stages.

### 5.4. Manufacturing Scale-Up and Functional Comparability

**Figure 3:**
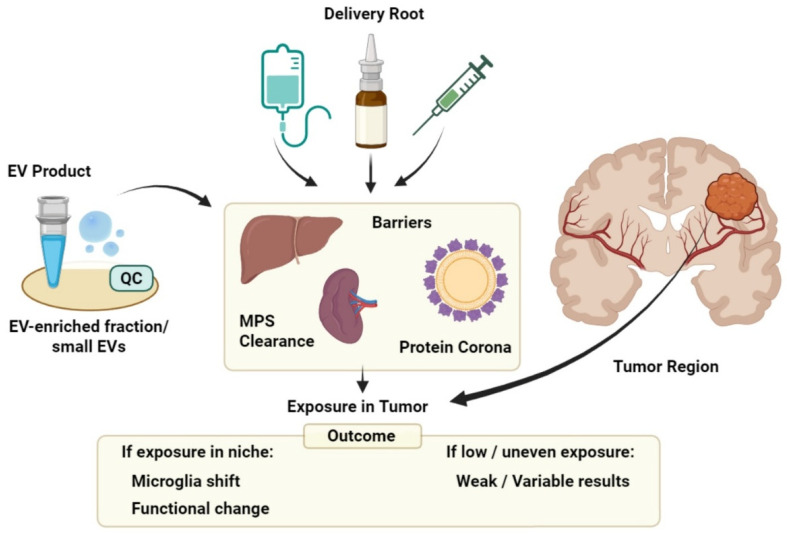
EV formulations (EV-enriched/small EVs; QC-verified) delivered by different administration routes encounter systemic barriers, including MPS clearance and protein corona formation, which together determine effective exposure within heterogeneous GBM tumor regions. Adequate niche exposure can drive a microglial state shift, whereas low or uneven exposure yields weak and variable outcomes.

The potential clinical development of EVs requires manufacturing scale-up, which introduces constraints that extend beyond yield and process efficiency. Changes in culture format, expansion strategy, or collection volume can alter the biological characteristics of EV preparations, even when isolation methods and nominal quality metrics remain unchanged. So, scale-up should be considered as a real biological variable that influences the functional outcome (Figure 3).

Batch-to-batch variability represents a central challenge in this context, with EV cargo composition reflecting the physiological state of the EV producer cells at the time of release, which can drift along cell passage number, culture density, and environmental stress. These shifts may not be detectable through standard particle or protein measurements, yet they can substantially affect the capacity of EVs to modulate microglial response. Without functional comparability across batches, apparent inconsistencies in biological activity are difficult to interpret. Additionally, culture conditions to scale-up production, involving process modifications to accommodate larger volumes or longer production runs, influence EV stability and content according to media formulation, oxygen tension, or conditioning duration. This can cause bias in cargo enrichment and alter downstream effects, introducing systematic divergence between early proof-of-concept material and later-generation EV preparations.

Comparability across EV production platforms further complicates translation: different expansion or collection systems may satisfy similar descriptive criteria while exhibiting divergent biological behavior. Without side-by-side evaluation using state-sensitive potency assays, platform-related effects can be misattributed to experimental noise or biological variability rather than manufacturing-dependent differences.

Addressing these challenges will require incorporating large-scale EV production comparisons as an active design objective, rather than a retrospective check. Early integration of functional benchmarks, together with controlled evaluation of scale-dependent changes, can strengthen confidence that observed microglial effects are intrinsic to the EV product rather than artifacts of the production process. This approach supports more reliable progression from exploratory studies toward translational development.

### 5.5. Developmental and Regulatory Perspective

As EV-based approaches move beyond proof of concept, interpretive rigor becomes as important as biological innovation. Early stage studies often prioritize demonstration of activity, yet translational progression requires a shift toward a disciplined definition of what constitutes a viable immunomodulatory product. In this context, developmental logic functions as a filter, distinguishing exploratory signals from effects that are robust enough to justify further investment.

In a translational framework, it is required that product identity is clearly defined to ensure that biological effects can be attributed to the intended EV population. Moreover, efficacy assessment requires the definition of a consistent mechanism and state-sensitive readouts that reflect durable microglial modulation. Dose must be interpretable in relation to effective exposure, and delivery assumptions must be supported rather than inferred. Without alignment across these dimensions, apparent activity remains difficult to generalize or reproduce.

From a regulatory standpoint, this framework does not impose rigidity but rather creates clarity. Predefined decision criteria reduce ambiguity around negative results and prevent premature escalation of EV candidates whose effects depend on poorly controlled variables. Products that satisfy these criteria gain credibility, as their observed activity can be linked to defined properties rather than experimental context.

Importantly, disciplined development does not constrain innovation but protects it. By enforcing equivalence, interpretability, and defined exposure parameters, translational standards help ensure that promising EV-based immunomodulatory strategies are advanced on the basis of reproducible biology rather than optimistic inference. In a complex disease environment, as occurs in GBM, this discipline is not optional but essential for meaningful progress.

## 6. Mechanistic Logic of Microglia Reprogramming by Stem Cell-Derived Factors

Microglia reprogramming indicates a tumor- or therapy-driven shift in the functional state of resident CNS microglia, and not a mere on/off polarization event. In detail, this refers to coordinated changes in transcriptional programs, signaling outputs, and effector functions that move microglia along a continuum from a homeostatic toward a glioma-conditioned state that can be therapeutically redirected. Importantly, this concept should not be interpreted as automatically interchangeable with global TAM modulation. Stronger evidence for microglia-specific reprogramming comes from studies that preserve lineage-aware analysis, distinguishing resident microglia from infiltrating macrophages, using markers such as P2RY12, TMEM119, and FCRLS for microglia and CD49d/Itga4 for blood-derived macrophages, together with spatial or single-cell transcriptomic profiling [191,192,193]. Accordingly, in the context of GBM, the most rigorous interpretation does not consider all observed myeloid changes as related to microglia, but that microglia-specific reprogramming can only be claimed when state changes are directly demonstrated within the resident microglial compartment, rather than inferred from pooled TAM populations [194].

In this complex context, both stem cell-derived secretomes and EVs, should be considered as tunable inputs to modulate microglial activity, rather than as generic “pro-” or “anti-inflammatory” stimuli. For GBM-oriented studies, the practical mechanistic question is whether these inputs modulate microglia’s ability to integrate concurrent cues in the niche, without revisiting the upstream pathway biology covered above.

### 6.1. miRNA-Mediated Biasing of Microglial Regulatory Programs

EV-mediated miRNA transfer can influence microglial transcriptional control because miRNAs act upstream of broad effector programs [195]. When miRNA cargo is functionally delivered, it can attenuate or rebalance signaling intermediates and transcriptional regulators, thereby modulating the activation thresholds at which specific response programs become self-sustaining [196]. In this framework, microglial reprogramming does not rely on a single miRNA to define a specific phenotype; rather, it involves miRNA cargo that biases the regulatory landscape, such that identical inputs can produce distinct downstream responses.

miR-124 is often reported to be linked to reduced engagement of inflammatory transcriptional programs, within CNS immune settings [197,198]. Rather than treating it as a universal anti-inflammatory switch, a more conservative interpretation is that miR-124 can modulate upstream regulators in ways that reduce the persistence of maladaptive activation patterns under chronic stimulation [199,200].

miR-146a represents a complementary control strategy and it is well established as part of endogenous negative feedback control in inflammatory signaling and can limit pathway amplification by targeting key propagation nodes [201,202]. In tumor-conditioned contexts, tightening this feedback can reduce the dominance of self-reinforcing signaling and contribute to a recalibrated inflammatory setpoint, with outcomes depending on the surrounding niche [203].

Together, these two examples support a useful working principle for EV engineering and interpretation: miRNA cargo is most informative when it is linked to regulatory behavior (thresholding, feedback strength, and stability of transcriptional states) rather than to shifts in a limited marker panel.

### 6.2. Context-Dependent Stabilization of Microglial State

For stem cell-derived interventions, miRNA-driven regulation and soluble factor signaling are typically best interpreted as coupled inputs: one layer biases intracellular control, while another provides directional pressure. Whether the resulting shift persists depends on how strongly local constraints favor reversion toward the baseline tumor-conditioned program.

Accordingly, reprogramming in GBM is more accurately defined as constraint-limited plasticity than as free switching between states. Interventions can look active yet remain shallow if they provide direction without changing the stability of the underlying program, or if they return regulators without sufficient contextual drive. The most plausible durable shifts are those in which these layers converge on the same functional outcome.

**Figure 4:**
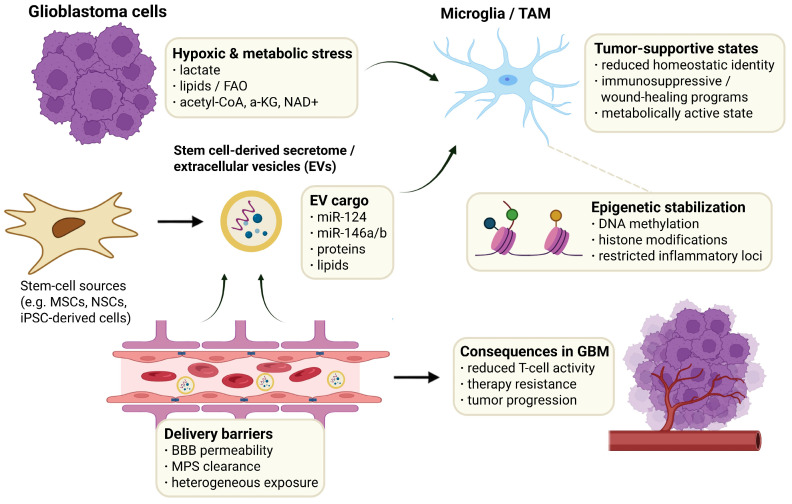
Stem cell-derived EV/secretome regulation of microglia/TAM states in GBM.

GBM-derived hypoxic and metabolic stress promotes tumor-supportive microglia/TAM states through lactate, lipid metabolism, FAO, and metabolic cofactors linked to epigenetic stabilization. Stem cell-derived secretomes and EVs may modulate these states through regulatory cargo, including miR-124, miR-146a/b, proteins, and lipids. However, their effect depends on delivery barriers such as BBB permeability, MPS clearance, and heterogeneous tumor exposure, which can influence the extent of microglial modulation and downstream GBM progression (Figure 4).

## 7. Preclinical Evidence and Translational Relevance

Preclinical studies emphasize this line of reasoning in a biological context. Most mechanistic claims come from controlled systems where microglia are exposed to glioma-associated cues and then treated with stem cell secretome products or EV-enriched fractions. These models are useful because they reduce complexity, allowing a clear definition of the role of the actors in the biological response.

Microglia/GBM cell co-cultures are widely used for this purpose, although the protocol used may introduce bias in the observed results. Transwell systems emphasize soluble factor crosstalk. Direct contact setups add adhesion and matrix-dependent effects that can change baseline activation. These choices will shape the “reprogramming” occurring even before treatment begins, which is one reason why different studies can report different patterns even when the headline intervention seems similar (Table 3).

In these platforms, EV/secretome exposure is typically evaluated using two layers of readouts (Table 4). First are molecular or descriptive endpoints: changes in inflammatory and immunoregulatory outputs, shifts in antigen presentation associated features, or changes in transcriptional programs aligned with the non-binary microglial states described earlier. These signals help define directionality, but they are not sufficient on their own.

The second layer is a functional readout, which is more informative when the goal is possible biological relevance rather than description. Phagocytosis assays can test whether microglia become more competent at engulfment in glioma-conditioned contexts. Cytokine balance measurement may be an index of whether output is pushed toward a more immune permissive profile. Invasion-linked assays can prove whether microglia remain supportive of GBM infiltration behavior under the same conditions. None of these assays is perfect per se, but together they help avoid overinterpreting marker changes as durable state shifts [33,96].

Ex vivo and 3D cultures can add a more realistic representation of the tissue/tumor interaction by preserving tissue structure and multicellular context. They narrow the gap between simplified co-cultures and vivo systems, while still allowing mechanistic testing under defined conditions. The trade-off is that these systems are harder to standardize and often have limited windows for stable observation, which makes persistence-based interpretation more challenging. Overall, in vitro and ex vivo evidence is most convincing when it links EV/secretome exposure to a functional change, rather than merely assessing marker shifts.

### 7.1. In Vivo Glioma Models

In vivo studies allow the testing of the potential microglial reprogramming under a more realistic biological complexity. However, they also introduce the largest source of interpretive uncertainty, whether the intervention engages the intended compartment in a way that supports clean conclusions. For that reason, in vivo findings are most useful when they are analyzed alongside the delivery and exposure constraints discussed earlier, rather than being treated as standalone validation.

Evidence for microglia-specific reprogramming can be provided with high validity when resident microglia are clearly distinguished from infiltrating macrophages. In this way, the observed changes can be exclusively referred to the resident compartment itself. Accordingly, the most convincing studies are those that combine lineage-aware markers, spatial or single-cell analyses, and functional readouts to show that resident microglia undergo a defined transcriptional or phenotypic shift under glioma-associated or therapeutic pressure. By contrast, changes measured only in pooled TAM fractions, bulk immune populations, or tumor-level outcomes should be interpreted as broader myeloid modulation unless microglial specificity is directly shown [51,204,205,206].

Mouse glioma models have been used to examine EV or secretome delivery in parallel with microglial readouts and tumor-level outcomes. The typical endpoints include changes in myeloid transcriptional programs, shifts in immune marker patterns, and macroscopic effects on tumor growth or survival. These data can support the possible efficacy of the treatment, but mechanistic attribution still requires care [207].

A recurring challenge is the heterogeneity of tumor-associated myeloid populations. Even when the experimental aim is to reprogram microglia, observed signals can be influenced by infiltrating macrophages or by temporal shifts in the relative composition of myeloid subsets. This does not invalidate the findings, but it underscores that the most robust studies are those that preserve lineage-resolved analyses and avoid collapsing distinct compartments into a single, aggregated TAM response.

Tumor-level outcomes are important; however, they are not a direct evidence of microglia-mediated mechanisms. Effects on survival or tumor growth may instead reflect multiple layers of underlying biology. Conversely, the absence of tumor control does not necessarily indicate unchanged microglial function, but rather that any shift was insufficient in magnitude or duration to affect the overall tumor trajectory [195].

### 7.2. Translational Relevance and Limitations

Even in the presence of promising preclinical data, translation remains non-trivial for several reasons. Species difference is one of the most obvious reasons. Mouse and human microglia share developmental logic but differ in baseline programs and responsiveness, which matters when the goal is to bias regulatory behavior rather than merely induce an “on/off” phenotype [208].

Another major limitation is the lack of consistency in how reprogramming is defined across studies. Some studies rely on small marker panels, others emphasize cytokine shifts, use transcriptomic signatures, or functional assays. Without convergence on state relevant and comparable endpoints, the field risks producing results that are difficult to reconcile across laboratories, even when the underlying biology is not contradictory [209,210].

Finally, GBM itself applies strong selective pressure. The tumor niche is heterogeneous, adaptive, and actively immunosuppressive. Preclinical findings therefore need to be considered as evidence of plausibility under defined conditions, rather than as guarantees of clinical efficacy. The translational value of these studies is that they narrow the hypothesis space: they indicate which kinds of inputs can shift microglial behavior, under what experimental constraints, and with what limits.

## 8. Future Directions for Precision Microglia Reprogramming in Glioblastoma

Progress in this field will not come by adding additional descriptive EV studies, but from building interventions that answer one clear question at a time. The next phase should treat microglia reprogramming as an engineering problem inside a hostile niche, where the key metric is not novelty but reproducibility under realistic constraints.

Engineered EVs are valuable because they enable a shift from associative observations to hypothesis-driven design. Rather than asking whether EVs exert an effect, the field can investigate whether a defined cargo or specific surface feature directs microglial behavior in the intended direction under glioma-conditioned pressure. This approach also enhances experimental tractability, as the intervention introduces a controllable variable that can be systematically modified, tuned, and compared.

A practical strategy is to use engineered EVs to isolate causal mechanisms. When a specific payload is hypothesized to bias microglia toward a less tumor-supportive phenotype, EVs function as a controlled input rather than a heterogeneous mixture. Even unsuccessful engineered constructs can be informative, as they help exclude factors that do not drive the system under GBM-like conditions. Combination therapy will likely be required for GBM; however, combinations should be based on complementary mechanisms rather than presumed synergy. Microglia-directed modulation may be most effective when paired with interventions that alter the tumor microenvironmental pressures maintaining myeloid cells in a tumor-supportive state.

A key future direction is the rational design of combinations: therapies should be selected to address distinct biological constraints, rather than layering multiple immune interventions in the hope of amplifying the response. Some combinations may be synergistic, others antagonistic, and some may simply introduce interpretative noise. Only a rationale-driven design framework will allow meaningful insights to be derived, regardless of outcome.

### 8.1. Precision Testing of Microglia Reprogramming in Human-Relevant Systems

A major future step in this research field is the expansion of the use of human-relevant systems, retaining the molecular and cellular crosstalk that shapes microglial behavior in tumors. Co-culture complexity is critical in this context. Models incorporating glioma cells together with myeloid populations in a structured setting may enable future studies to assess whether an intervention remains effective when signals interact, rather than when they are applied individually.

Organoids, engineered co-cultures, and patient-derived cultures are useful because they can retain subtly regulated mechanisms. If a reprogramming strategy is effective only in simplified systems, it is preferable to establish this early, before advancing the treatment.

Moreover, the field does not need a long list of candidate cargos, but fewer, sharper hypotheses that can be compared across studies. Future progress will be faster if studies converge on a small number of defined experimental questions and build around them, rather than generating broad catalogs of promising effects that are difficult to reconcile.

In practice, this entails: (i) selecting a specific microglial function to target; (ii) designing an intervention around that target; and (iii) testing it in systems capable of falsifying the hypothesis. Such an approach limits overinterpretation and helps identify which strategies warrant progression to more complex experimental settings.

### 8.2. AI and Multi-Omics Integration

A final gap in the current landscape is the lack of integrative frameworks to reconcile the complexity of secretome and EV cargo inputs with the layered regulatory behavior of microglia. As datasets expand beyond single readouts, AI-assisted and multi-omics approaches offer a way to move from descriptive association toward systems-level understanding. The value of these tools is not predictive for their own sake, but within structured hypothesis generation, identifying which combinations of signals are most likely to shift microglial regulatory states under tumor-conditioned constraints.

Multi-omics integration, including transcriptomic, epigenomic, metabolic, and proteomic profiles, can help in the discrimination of merely correlative signals from those consistently converging on shared control nodes. Computational modeling becomes a way to prioritize experiments rather than replace them. Network-based analyses can suggest which microglial programs are most sensitive to modulation, which inputs are redundant, and where compensatory circuits are likely to blunt intervention.

More specifically, integrative computational approaches could include gene regulatory network inference, pathway-level modeling, and network-based prioritization of key control nodes within glioma-associated myeloid signaling programs. Such frameworks may help identify regulatory pathways whose perturbation is most likely to shift myeloid cells away from tumor-supportive states, while also revealing compensatory circuits that may limit therapeutic efficacy. In this context, integrating epigenomic, transcriptomic, and proteomic data with network-based sensitivity analysis could provide a more systematic basis for prioritizing candidate targets for functional validation [211,212]. Importantly, such models are only as useful as the data they integrate, reinforcing the need for lineage-aware, state-relevant, and functionally anchored measurements.

AI-driven approaches are therefore best regarded as tools for mapping constraints when applied to well-designed experimental datasets. They can narrow the hypothesis space, reduce exploration noise, and guide the selection of interventions that warrant testing in more complex systems. When used in this way, computational integration does not introduce additional layers of notion, but it imposes discipline on an otherwise combinatorial problem, aligning experimental design with the goal of reproducible, mechanism-informed microglia reprogramming in GBM.

## 9. Conclusions

After decades of pharmacological, genetic, and molecular research, GBM is still a difficult tumor to treat and, inevitably, with very poor prognosis. This is not due to the lack of knowledge about the most relevant pathways involved in the proliferation and invasiveness of this tumor, which have been deeply characterized, but because the tumor resists interventions through the development of a heterogeneous ecosystem that rapidly adapts. Targeted approaches have repeatedly hit the limits of redundancy and intratumoral variation, and immune strategies have struggled in a tumor that is poorly immunogenic and dominated by immunosuppressive myeloid cells. In that setting, the state of the microenvironment is a primary determinant of the efficacy of therapy.

Microglia and other tumor-associated myeloid cells, which sit at the center of this problem, are plastic, but plasticity is not free. It is shaped and stabilized by tumor-derived cues, metabolic pressures, and epigenetic reinforcement that collectively bias the system toward tumor support. Stem cell-derived secretomes and EVs are therefore attractive not as direct anti-tumor agents, but as tunable inputs that can, in principle, shift the mechanisms that microglia use to integrate competing signals in the glioma niche, with miRNA possibly involved in bias control nodes and feedback behavior, while soluble factors apply directional pressure. EV identity is often operational, delivery and exposure are uneven across tumor regions, dose units are inconsistent, and microglial readouts can be confounded by mixed myeloid populations. For these reasons, the strongest message from current evidence is not that clinical translation is guaranteed, but that plausibility can be demonstrated under clearly defined constraints.

A practical translational framework emerges directly from these observations. Claims of reprogramming are most compelling when a defined product can be linked to confirmed exposure, assessed using mechanism-consistent and state-sensitive potency readouts, and anchored to functional outcomes that are relevant in glioma-conditioned contexts. Importantly, this approach should preserve lineage awareness, rather than collapsing distinct compartments into a single TAM signal. When these elements align, positive results become interpretable and negative results become useful rather than ambiguous.

Progress depends on precision studies: choosing a microglial function to target, designing an intervention around that target, and testing it in systems that can falsify the idea. Engineered EVs support this approach by turning cargo into a controlled variable. Combination strategies should be built around complementary roles instead of assumed synergy, and human-relevant models can expose fragile mechanisms before claims are escalated. Finally, AI-assisted and multi-omics integration can help prioritize which inputs and control nodes merit deeper testing. Together, these directions point to a realistic path forward, one where microglia reprogramming is developed as an exposure-aware, mechanism-guided strategy that can remain credible inside the constraints of the glioblastoma microenvironment.

## Figures and Tables

**Table 1:** Resident microglia vs. infiltrating macrophages (BMDM-derived) in GBM.

Feature	Resident Microglia	Infiltrating Macrophages (BMDM-Derived)
Origin	Yolk sac-derived; seed CNS before BBB closure	Blood-derived; recruited into tumor
Markers	P2ry12, Fcrls, Tmem119 (maintained by TGF-β)	High Itga4 (CD49d)
Tumor localization	Leading edge/infiltration zones	Perivascular and necrotic regions
Program	Homeostatic-primed spectrum; can show higher MHC-II and lipid metabolism genes	Immunosuppressive/wound-healing programs; higher cytokine phagocytosis and TCA cycle markers

**Table 2:** Stem cell secretome sources.

Source	Profile	Examples in Text	Key Limitation
MSC	Immunomodulatory (soluble + EV)	IDO/PGE2/NO; EV miRNAs miR-124, miR-146b	High variability (source/culture; EV cargo/yield)
NSC	Neurotrophic	NGF/BDNF/CNTF/GDNF; EV miR-124-3p	Different profile than MSCs
iPSC-derived	Scalable but variable	Donor-dependent secretome; iPSC-EVs reported anti-inflammatory/pro-vascularization	Standardization not established

**Table 3:** Biological and translational barriers shaping microglia-reprogramming strategies in GBM.

Barrier	Biological Meaning	Implication
Myeloid heterogeneity	TAMs include resident microglia and infiltrating macrophages	Analyze myeloid subsets separately
Non-binary activation states	TAMs occupy mixed activation states beyond M1/M2	Avoid single-marker M1/M2 interpretation
Metabolic constraint	Hypoxia, lactate, lipid metabolism, FAO, and TCA activity reinforce tumor-supportive myeloid programs	Test EV/secretome effects under GBM-like pressure
Epigenetic stabilization	Chromatin changes can maintain immunosuppressive programs	Assess durability, not only acute state changes
Uneven delivery/exposure	BBB permeability heterogeneity, clearance, and protein corona limit EV access	Confirm tumor-region exposure
Product variability	Source, culture, isolation, and scale-up alter EV/secretome composition	Use potency assays to compare products

**Table 4:** Preclinical models used to assess secretome/EV effects on microglia in GBM.

Model System	What It Captures	Main Limitation
Transwell co-culture	Soluble crosstalk	Model design shapes baseline “reprogramming” pattern
Direct-contact co-culture	Adhesion + matrix effects	Different setups can produce different outcomes
Ex vivo models	Tissue structure + multicellular context	Hard to standardize; limited stable observation window
In vivo glioma models	Full biological complexity + tumor outcomes	Attribution limited by exposure uncertainty and mixed myeloid populations

## Data Availability

No new data were created or analyzed in this study.
